# Effect of Prophylactic Central Lymph Node Dissection on Locoregional Recurrence in Patients with Papillary Thyroid Microcarcinoma

**DOI:** 10.1155/2021/8270622

**Published:** 2021-11-15

**Authors:** Peipei Yang, Jianming Li, Haoyu Jing, Qiyang Chen, Xinxin Song, Linxue Qian

**Affiliations:** ^1^Department of Ultrasound, Beijing Friendship Hospital, Capital Medical University, Yongan Road No. 95, Xicheng District, Beijing 100050, China; ^2^Department of Interventional Ultrasound, First Medical Center of Chinese People's Liberation Army, General Hospital, 28 Fuxing Road, Haidian District, Beijing 100853, China

## Abstract

There is a consensus that central compartment lymph node dissection or modified radical lateral neck dissection should be performed in papillary thyroid microcarcinoma (PTMC) patients with lymph node metastases. Prophylactic central lymph node dissection (PCLND) in patients with clinically node-negative (cN0) PTMC to reduce locoregional recurrence (LRR) rate and improve prognosis remains controversial. The present study aimed to analyze the effect of PCLND on LRR and postoperative complications of PTMC in cN0 patients. We reviewed a cohort of patients with cN0 PTMC who underwent surgery between January 1997 and October 2019. The patients were divided into the PCLND and no lymph node dissection (NLND) groups. Kaplan–Meier curves were constructed to estimate 15-year locoregional recurrence-free survival rate of the two groups, and the difference was compared by the log-rank test. Three Cox regression models were performed to evaluate the correlation between PCLND and LRR. All patients underwent thyroidectomy, and 25 patients developed LRR; of whom, 23 underwent PCLND at initial surgery and 2 went without lymph node dissection. Cox regression analysis showed that PCLND had no effect on LRR. Postoperative hematoma and permanent recurrent laryngeal nerve injury did not occur in the NLND group, and their incidences were 0.5% and 0.3% in the PCLND group, respectively. PCLND had no significant correlation with LRR in patients with cN0 PTMC, and the absolute benefit for PTMC was small.

## 1. Introduction

Papillary thyroid cancer (PTC) is the most common subtype of differentiated thyroid cancer and accounts for >80% of all thyroid cancers [1, 2]. The overall prognosis of patients with PTC is favorable, with a 10-year survival rate >90% [3, 4]. Papillary thyroid microcarcinoma (PTMC) is defined by the World Health Organization as PTC ≤1 cm in maximal diameter. In recent years, owing to the widespread application of high-frequency ultrasound and fine needle aspiration biopsy, the incidence of PTMC has increased sharply. PTMC has become the most common form of PTC, accounting for 80% of all cases [5].

Although PTMC is considered to be an indolent tumor, the incidence of lymph node metastasis is as high as 24.1–64.1% [6–8]. Currently, thyroidectomy combined with regional lymph node dissection has become the consensus for the treatment of PTMC with clinically proven lymph node metastases. However, the need for prophylactic central lymph node dissection (PCLND) in PTMC patients without lymphatic metastasis remains controversial. So far, no convincing evidence has proven that PCLND significantly improves the prognosis, and guideline opinions differ widely on the best treatment for PTMC [9–11].

Therefore, we conducted this retrospective study to analyze the effect of PCLND on locoregional recurrence (LRR) and postoperative complications of patients with clinically node-negative (cN0) PTMC.

## 2. Methods

### 2.1. Study Population

We retrospectively analyzed a cohort of patients with cN0 PTMC who initially underwent surgery between January 1997 and October 2019 at Beijing Friendship Hospital, Capital Medical University.

### 2.2. Inclusion and Exclusion Criteria

All patients were evaluated preoperatively with physical examination and ultrasonography to assess cervical lymph node metastasis. Patients with no history of thyroid or neck surgery and no evidence of lymph node metastasis in preoperative imaging were enrolled. The exclusion criteria included other types of thyroid cancer (such as follicular thyroid cancer, medullary carcinoma, and metastatic cancer) and patients who underwent lymph node dissection other than PCLND. Patients with an interval between surgery and recurrence of <6 months were considered to have persistent disease and were also excluded.

### 2.3. Data Collection

Detailed information was collected from the medical records including age, sex, type of operative procedure and lymph node dissection, operative complications, tumor size, multifocality, capsule invasion, extrathyroidal extension (ETE), and postoperative occult neck lymph node metastases. Multifocality referred to the presence of multiple tumor foci in the thyroid, and only the maximum diameter of the largest foci was analyzed. ETE was defined as tumor penetration of the thyroid capsule, with invasion of surrounding soft tissues and organs. Patients were grouped according to type of lymph node dissection: patients who underwent thyroidectomy combined with PCLND were designated as the PCLND group, whereas those who underwent thyroidectomy alone were designated as the NLND group.

### 2.4. Definition of Clinical Outcomes

After initial surgery, all patients were followed up with physical examination, neck ultrasonography, and thyroid function testing every 3–6 months. Fine needle aspiration biopsy was performed for diagnosis of suspicious thyroid nodules or metastatic lymph nodes. The endpoint of this study was LRR or distant metastases. LRR included local recurrence (recurrence in the remnant thyroid tissue or thyroid bed) and regional lymph node recurrence [12, 13]. Major postoperative complications were recorded, including hematoma, parathyroid hypothyroidism, and recurrent laryngeal nerve injury. Temporary parathyroid hypothyroidism was defined as blood calcium levels <8 mg/dL within 6 months, and temporary recurrent laryngeal nerve injury was defined as hoarseness and vocal cord paralysis [6, 14]. Impaired parathyroid and recurrent laryngeal nerve function that persisted for >6 months was defined as permanent hypothyroidism or permanent recurrent laryngeal nerve injury [14]. Laryngoscopy was performed in all patients to confirm vocal cord mobility before and after surgery.

### 2.5. Statistical Analysis

SPSS version 20.0 statistical software (SPSS Inc., Chicago, IL, USA) was used to analyze the data. Continuous variables were presented as means and standard deviations and categorical variables as percentages and instances. Comparisons between groups were performed by Student's *t*-test or chi-square test. Kaplan–Meier curves were constructed to estimate 15-year locoregional recurrence-free survival (LRFS) rate, and the rates in the two groups were compared by the log-rank test. Cox regression analysis was performed with three models: 1, an unadjusted model was established by one-on-one association of PCLND and LRR; 2, a multivariate model adjusted for age and sex; and 3, a multivariate model adjusted for all factors (such as age, sex, type of operative procedure, operative complications, tumor size, multifocality, capsule invasion, ETE, and occult central lymph node metastases). Association of PCLND with LRR was analyzed via the three models. *P* < 0.05 was considered to be statistically significant.

### 2.6. Ethical Considerations

The study was approved by the Research Ethics Committee of Beijing Friendship Hospital (No. 2019-P2-159-01). Owing to the retrospective nature of the research, the need for informed consent was waived.

## 3. Results

The demographics and pathological features of the patients are given in Table 1.

A cohort of 1584 patients was divided into 2 groups according to PCLND: 1484 underwent thyroidectomy plus PCLND and 100 underwent thyroidectomy alone. The mean ages of the two groups were 46.92 ± 11.72 and 48.05 ± 10.89 years, respectively (*P* > 0.05). The average maximum diameter of the lesions was 0.59 ± 0.23 cm in the PCLND group and 0.56 ± 0.31 cm in the NLND group. In the PCLND group, 678 patients (45.7%) underwent total thyroidectomy (TT), 122 (8.2%) underwent subtotal thyroidectomy, and 684 (46.1%) underwent unilateral lobectomy. This differed significantly from the NLND group: 15 patients underwent TT (15.0%), 54 subtotal thyroidectomy (54.0%), and 31 unilateral lobectomy (46.1%) (*P* < 0.001). There were significantly more patients with capsular invasion in the PCLND group than in the NLND group (33.0% vs. 10.0%; *P* < 0.001). Other demographic and clinicopathological features such as age, tumor diameter, multifocality, and ETE were compared between the two groups (Table 2), and no significant differences existed. Unexpectedly, occult central lymph node metastases were discovered incidentally in the removed perithyroidal lymph nodes in 4 cases in the NLND group (Table 2).

**Figure 1:**
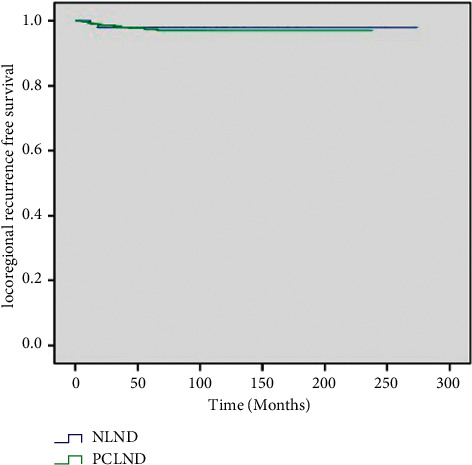
Kaplan–Meier curve of local recurrence-free survival. PCLND, prophylactic central lymph node dissection; NLND, no lymph node dissection.

Long-term follow-up showed that LRR occurred in 25 patients, including 23 in the PCLND group and 2 in the NLND group. Kaplan–Meier curves showed that the cumulative 15-year LRFS rates were 96.9% and 97.9% in the PCLND and NLND groups, respectively. The overall LRFS rates in the two groups showed no significant difference (*P*=0.795) (Figure 1). There was no significant difference in the complication rate between the two groups (*P*=0.765). However, while postoperative hematoma and permanent recurrent laryngeal nerve injury did not occur in the NLND group, their incidences were 0.5% and 0.3% in the PCLND group, respectively (Table 3).

Three Cox regression models were performed to evaluate the correlation between PCLND and LRR. In the first model, only the variable of PCLND was analyzed, which showed that PCLND had no effect on LRR. In the second model, multivariate analysis was adjusted by age and sex, which showed that PCLND had no significant correlation with LRR. In the third model, all factors were adjusted in multivariate analysis, but PCLND still had no significant correlation with LRR (Table 4).

No distant recurrence and death was occurred during the follow-up period.

## 4. Discussion

In this study, three Cox regression analysis models (1, unadjusted model; 2, age–sex model; and 3, multivariate model) were constructed to assess the correlation between PCLND and LRR. All of the regression analyses suggested that PCLND had no significant correlation with LRR (*P* > 0.05). Kaplan–Meier curves estimated the cumulative 15-year LRFS rates and revealed no significant differences in overall LRFS rates between the PCLND and NLND groups. These results are in accordance with previous research [8, 15, 16].

Wada et al. evaluated the clinical significance of PCLND in the prognosis of patients with PTMC. A total of 235 patients without palpable nodes underwent PCLND, and 155 patients whose PTMC was discovered incidentally underwent thyroidectomy alone. The recurrence rate was 0.43% in the prophylactic group and 0.65% in the control group, but there was no significant difference in the recurrence rate when compared between the two groups [8]. A meta-analysis of 1246 patients showed that there was no significant difference in the local recurrence rates between the thyroidectomy + PCLND and thyroidectomy alone groups [15]. Thus, PCLND cannot greatly reduce LRR rate in patients with cN0 PTMC. Additionally, the previous studies revealed that the disease-specific mortality of PTMC was <1%, the local recurrence rate was 2–6%, and the distant recurrence rate was 1-2% [17, 18]. Therefore, the excellent prognosis of PTMC may be more related to the indolent nature of the tumor than the therapeutic effect. Similar clinical results were observed in two prospective studies from Japan on active surveillance of low-risk PTMC patients [19, 20]. Sugitani et al. reported that delayed surgery does not influence prognosis. No recurrence was observed after surgery in patients whose tumors increased in size or metastasized to lymph nodes after 1–12 years of nonsurgical observation [20]. Furthermore, PCLND does not bring any survival benefit to patients with occult skip lymph node metastasis. PTMC usually metastasizes to the central lymph nodes, whereas skip metastasis of lateral cervical lymph nodes occurred in 3.7–44.5% of patients [6]. PCLND cannot eradicate all lesions and reduce the recurrence rate in lateral cervical lymph nodes [21].

Several studies have supported the concept that PCLND should be routinely performed in PTMC patients because metastatic lymph nodes were found in only 28.2–64.2% of cases by preoperative clinical palpation and ultrasound (US) examination [22–24]. However, occult central lymph node metastases were found in 31–66% of patients who had no evidence of lymph node metastases on clinical or US examination [8, 25–27]. Thus, supporters have argued that PCLND can facilitate complete resection of occult central lymph node metastases and reduce the risk of LRR, ultimately improving disease-free survival [28].

Conversely, numerous studies have indicated that occult lymph node metastasis does not affect prognosis of PTMC patients. The American Joint Commission on Cancer TNM staging guidelines (8th edition) suggest that the prognosis of patients with occult nodal disease after PCLND does not differ significantly from that in patients without occult metastatic lymph nodes [29]. Shaha et al. also noted that the clinical recurrence rate ranged from 10% to 20%, whereas the incidence of occult lymph node metastasis was 70–80% if PCLND was applied routinely to PTMC patients [30]. Eventhough PTMC exhibits a high rate of occult lymph node metastasis, there are few metastatic lymph nodes with clinical significance.

In our series, 475 patients classified as cN0 by clinical or US examination had occult lymph node metastases on pathological examination. Four of the patients were in the NLND group because the lymph nodes attached to the thyroid gland were unknowingly removed during the operation. It can be inferred that patients in the NLND group had a higher rate of occult lymph node metastasis, based on the results of previous studies. However, Cox regression analysis did not show any significant difference in LRR rates between the PCLND and NLND groups (*P* > 0.05). Viola et al. analyzed patients with cN0 PTMC including 88 with TT and 93 with TT + PCLND [31]. Histopathology detected occult lymph node metastasis in 46% of patients. However, the proportion of patients with disease-free survival and biochemical/structural recurrence was similar after a mean follow-up of 59 ± 7 months, but the difference between the two groups was not significant. These results confirmed that occult lymph node metastasis has no impact on prognosis.

In the present series, no postoperative hematoma and permanent recurrent laryngeal nerve injury occurred in the NLND group, whereas the respective rates in the PCLND group were up to 0.5% and 0.3%. In this study, it was impossible to compare the postoperative complications in patients undergoing different surgical procedures. However, several studies have reported that PCLND is associated with a higher risk of postoperative complications [32–35]. Lee et al. compared the recurrence rate of patients with TT alone and TT + PCLND, and there were no significant differences between the groups (3.9% vs. 3.3%, *P* > 0.05) [36]. In contrast, the incidence of complications was significantly higher in the TT + PCLND group than in the TT alone group, especially the incidence of transient hypocalcemia (*P* < 0.05). Two recent studies reported that the risk of hypocalcemia in patients treated with TT + PCLND was 2.0–2.7 times higher than that of TT alone. The lower parathyroid glands were injured accidentally during the operation because of their close proximity to the central lymph nodes [37, 38]. David et al. reported that the incidence of permanent hypoparathyroidism and permanent recurrent laryngeal nerve injury was 1-2% and 0–5.5% in patients with TT alone, but when combined with PCLND, the rates were 0–14.3% and 0–5.7%, respectively [13].

Additionally, postoperative complications are clearly related to the experience of the surgeon, and the risk is increased when the operation is performed by inexperienced thyroid surgeons [39]. Furthermore, when compared with TT alone, the incidence of hypoparathyroidism and recurrent laryngeal nerve injury in patients with TT + PCLND is still higher, eventhough the operation is performed by high-volume thyroid surgeons [40].

This study had several limitations. First, PTMC generally has a good prognosis. No distant metastasis and disease-specific mortality were found during follow-up, and the recurrence rate may have been underestimated. Second, the number of patients without PCLND was small, and increasing the study population in the NLND group in future studies would help to strengthen the results. Finally, since few patients were treated with radioactive iodine-131 therapy postoperatively, this situation was not analyzed in detail.

## 5. Conclusions

In patients with cN0 PTMC, PCLND has no significant correlation with LRR, and even if there is an incidence of occult lymph node metastasis, prognosis is usually not affected. Our study indicates that the absolute benefit of PCLND for patients with cN0 PTMC is small.

## Figures and Tables

**Table 1:** Baseline characteristics of patients (*N* = 1584).

	*N* (%)
Age	46.99 ± 11.66
Diameter	0.59 ± 0.24
Gender	
Males	339 (21.4%)
Females	1245 (78.6%)
LND	
No	100 (6.3%)
PCLND	1484 (93.7%)
Surgery	
TT	693 (43.8%)
ST	176 (11.1%)
LT	715 (45.1%)
Multifocality	
No	1181 (74.6%)
Yes	403 (25.4%)
Capsule invasion	
No	1084 (68.4%)
Yes	500 (31.6%)
ETE	
No	1548 (97.7%)
Yes	36 (2.3%)
Occult central lymph node metastases	
No	1109 (70.0%)
Yes	475 (30.0%)

LND, lymph node dissection; PCLND, prophylactic central lymph node dissection; TT, total thyroidectomy; ST, subtotal thyroidectomy; TL, thyroid lobectomy; ETE, extrathyroidal extension.

**Table 2:** Comparison of clinical and pathological features between PCLND and NLND groups.

	PCLND	NLND	*P* value
	(*N* = 1484)	(*N* = 100)	
Age	46.92 ± 11.72	48.05 ± 10.89	0.350
Diameter	0.59 ± 0.23	0.56 ± 0.31	0.291
Gender			0.035
Males	326 (22.0%)	13 (13.0%)	
Females	1158 (78.0%)	87 (87.0%)	
Surgery			<0.001^*∗*^
TT	678 (45.7%)	15 (15.0%)	
ST	122 (8.2%)	54 (54.0%)	
LT	684 (46.1%)	31 (31.0%)	
Multifocality			0.122
No	1100 (74.1%)	81 (82.0%)	
Yes	384 (25.9%)	19 (18.0%)	
Capsule invasion			<0.001^*∗*^
No	994 (67.0%)	90 (90.0%)	
Yes	490 (33.0%)	10 (10.0%)	
ETE			0.253
No	1452 (97.8%)	96 (96.0%)	
Yes	32 (2.2%)	4 (4.0%)	
Occult central lymph node metastases			<0.001^*∗*^
No	1013 (68.3%)	96 (96.0%)	
Yes	471 (31.7%)	4 (4.0%)	

PCLND, prophylactic central lymph node dissection; NLND, no lymph node dissection; TT, total thyroidectomy; ST, subtotal thyroidectomy; TL, thyroid lobectomy; ETE, extrathyroidal extension.

**Table 3:** Postoperative complications.

	PCLND	NLND	*P* value
	*N* = 1487	*N* = 100	
Postoperative complications			0.765
Hematoma	7 (0.5%)	0	
Temporary parathyroid hypothyroidism	96 (6.5%)	6(6.0%)	
Temporary recurrent laryngeal nerve injury	55 (3.7%)	4(4.0%)	
Permanent recurrent laryngeal nerve injury	5 (0.3%)	0	

PCLND, prophylactic central lymph node dissection; NLND, no lymph node dissection.

**Table 4:** Cox regression models of association between PCLND and LRR.

	Model 1		Model 2		Model 3	
	Hazard ratio	*P* value	Hazard ratio	*P* value	Hazard ratio	*P* value
PCLND	0.911 (0.214–3.885)	0.900	0.873 (0.204–3.734)	0.855	0.876 (0.177–4.334)	0.872
Age	—	—	0.976 (0.943–1.011)	0.175	0.990 (0.956–1.026)	0.596
Gender	—	—	0.854 (0.339–2.1507)	0.738	1.000 (0.391–2.563)	0.999
Surgery						
TT	—	—	—	—	1	
ST	—	—	—	—	0.601 (0.233–1.550)	0.292
LT	—	—	—	—	0.839 (0.244–2.879)	0.780
Diameter	—	—	—	—	3.400 (0.607–19.037)	0.164
Multifocality	—	—	—	—	0.549 (0.179–1.680)	0.293
Capsule invasion	—	—	—	—	0.619 (0.233–1.645)	0.337
ETE	—	—	—	—	1.958 (0.246–15.607)	0.526
Occult central lymph node metastases					2.200 (0.922–5.248)	0.075

PCLND, prophylactic central lymph node dissection; LRR, locoregional recurrence; TT, total thyroidectomy; ST, subtotal thyroidectomy; TL, thyroid lobectomy; ETE, extrathyroidal extension; Model 1, unadjusted model was established by one-on-one association of PCLND and regional recurrence; Model 2, model adjusted for age and sex; Model 3, multivariate model adjusted for all factors (such as age, sex, type of operative procedure, operative complications, tumor size, multifocality, capsule invasion, ETE, and occult central lymph node metastases).

## Data Availability

The data used to support the findings of this study are available from the corresponding author upon request.
